# The tripartite quantum-memory-assisted entropic uncertainty relation and upper bound on shareability of quantum discord

**DOI:** 10.1038/s41598-022-08098-z

**Published:** 2022-03-08

**Authors:** Hazhir Dolatkhah, Abolhassan Mohammadi, Soroush Haseli

**Affiliations:** 1 RCQI, Institute of physics, Slovak Academy of Sciences, Dúbravská Cesta 9, 84511 Bratislava, Slovakia; 2 Department of Physics, University of Kurdistan, Sanandaj, P. O. Box 66177-15175, Iran; 3 Faculty of Physics, Urmia University of Technology, Urmia, Iran

**Keywords:** Quantum information, Quantum mechanics

## Abstract

Quantum discord and quantum uncertainty are two important features of the quantum world. In this work, the relation between entropic uncertainty relation and the shareability of quantum discord is studied. By using tripartite quantum-memory-assisted entropic uncertainty relation, an upper bound for the shareability of quantum discord among different parties of a composite system is obtained. It is also shown that, for a specific class of tripartite states, the obtained relation could be expressed as monogamy of quantum discord. Moreover, it is illustrated that the relation could be generalized and an upper bound for the shareability of quantum discord for multipartite states is derived.

## Introduction

The uncertainty principle plays a crucial role in the field of quantum mechanics and it is known to be one of the fundamental concepts of the quantum world^1^. In quantum information theory, the uncertainty principle could be expressed in terms of the Shannon entropy. The entropy was used by Deutsch, as a criterion of uncertainty, which led to the formulation of the most famous form of the entropic uncertainty relation (EUR)^2^. The Deutsch’s uncertainty bound was modified by Kraus^3^, and a year later, it was proved by Maassen and Uffink^4^. The relation states that for two incompatible observables *X* and *Z*, the following EUR will hold

1 $$\begin{aligned} H(X) + H(Z) \ge \log _2{1 \over c} \equiv q_{MU} , \end{aligned}$$

in which $H(Q) = - \Sigma _k p_k \log _2p_k$ is the Shannon entropy of the measurable $Q \in \{X,Z\}$, $p_k$ stands for the probability of the outcome *k*, and the parameter *c* is defined as $c = max_{\{{\mathbb {X}},{\mathbb {Z}}\}} |\langle x_i| z_j \rangle |^2$, where ${\mathbb {X}}=\{ |x_i\rangle \}$ and ${\mathbb {Z}}=\{ |z_j\rangle \}$ are the eigenstates of the observables *X* and *Z*, respectively. Also, $q_{MU}$ is addressed as the incompatibility measure.

Expanding and modifying the relation is one of the main purposes in the field of quantum information, which is being pursued by many researchers^5–28^. In^5^, it was found that using the memory particle, the entropic uncertainty could be decreased. It resulted in a new uncertainty relation known as bipartite quantum-memory-assisted entropic uncertainty relation (QMA-EUR). The relation is read as

2 $$\begin{aligned} S(X|B) + S(Z|B) \ge q_{MU} + S(A|B), \end{aligned}$$

in which *S*(*A*|*B*) is the conditional von-Neumann entropy of $\rho _{AB}$, and $S({\mathscr {O}}|B) = S(\rho _{{\mathscr {O}}B}) - S(\rho _B), {\mathscr {O}} \in \{X,Z\}$ are the conditional von-Neumann entropies of the post-measurement states after measuring *X* and *Z* on the part *A*,

3 $$\begin{aligned} \rho _{XB}= & {} \sum _i \left( |x_i \rangle \langle x_i |_A \otimes I_B \right) \rho _{AB} \left( |x_i \rangle \langle x_i |_A \otimes I_B \right) , \end{aligned}$$

4 $$\begin{aligned} \rho _{ZB}= & {} \sum _j \left( |z_j \rangle \langle z_j |_A \otimes I_B \right) \rho _{AB} \left( |z_j \rangle \langle z_j |_A \otimes I_B \right) . \end{aligned}$$

The bipartite QMA-EUR could be extended to the tripartite QMA-EUR^5,6^, where the quantum memories are played by two extra particles *B* and *C*. In tripartite QMA-EUR, a quantum state $\rho _{ABC}$ is shared by Alice, Bob, and Charlie, so that Alice, Bob, and Charlie have access to parts *A*, *B*, and *C*, respectively. Then, Alice carries the measurement *X* or *Z* on her quantum system. Suppose that Alice measures *X*. Then, it is Bob’s job to minimize his uncertainty about *X*. On the other hand, if Alice measures *Z*, then it would be Charlie’s task to minimize his uncertainty about *Z*. The tripartite QMA-EUR is given by^5,6^,

5 $$\begin{aligned} S(X|B) + S(Z|C) \ge q_{MU}. \end{aligned}$$

Some efforts have been put into modifying and improving the bound presented in Eq. (5)^29,30^. In^30^, the lower bound of the tripartite QMA-EUR is improved by adding two additional terms to the lower bound of the relation as

6 $$\begin{aligned} S(X|B) + S(Z|C) \ge q_{MU} + {S(A|B) + S(A|C) \over 2} + \mathrm{max}\{0,\delta \}, \end{aligned}$$

where

$$\begin{aligned} \delta = {1 \over 2} [I(A:B)+I(A:C)] - [I(X:B)+I(Z:C)], \end{aligned}$$

in which *I*(*A* : *B*) and *I*(*P* : *B*) respectively are mutual information and Holevo quantity, given by

7 $$\begin{aligned} I(A:B)= & {} S(\rho _A) + S(\rho _B) - S(\rho _{AB}), \end{aligned}$$

8 $$\begin{aligned} I(P:B)= & {} S(\rho _B) - \sum _i p_i S(\rho _{B|i}), \end{aligned}$$

and the observable *P* is $P \in \{X,Z\}$. Note that, as the observable *P* on the part *A* is measured by Alice, the *i*-th outcome is obtained with probability $p_{i} = Tr_{AB} (\Pi _{i}^{A}\rho _{AB} \Pi _{i}^{A})$ and the part *B* is left in the corresponding state $S(\rho _{B|i}) =\frac{Tr_{A} (\Pi _{i}^{A}\rho _{AB} \Pi _{i}^{A})}{p_{i}}$. Recently, it is shown that this lower bound, Eq. (6), is tighter than the bounds that have been introduced^30,31^.

EURs and QMA-EURs with two observables are the topics we have discussed so far, but QMA-EURs can be generalized to more than two observables. This has been the main subject of many research studies and up to now, many QMA-EURs for more than two observables have been introduced^28,32–41^. For instance, new QMA-EUR for multipartite systems has been proposed in^42^, where the memory is divided into multiple parts, as follows

9 $$\begin{aligned} \sum _{m=1}^{N} S(M_m|X_m) \ge - \log _{2}(b) + {N-1 \over N} \sum _{m=1}^{N} S(A|X_m) +\mathrm{max}\{0,\delta ^{N} \} , \end{aligned}$$

in which

10 $$\begin{aligned} b=\max _{i_{N}} \left\{ \sum _{i_{2}\sim {i_{N-1}}} \max _{i_{1}} \left[ |\langle u^{1}_{i_{1}}|u^{2}_{i_{2}}\rangle |^{2} \right] \prod ^{N-1}_{m=2}|\langle u^{m}_{i_{m}}|u^{m+1}_{i_{m+1}}\rangle |^{2} \right\} , \end{aligned}$$

where $|u^{m}_{i_{m}}\rangle$ is the *i*-th eigenvector of $M_{m}$, and $\delta ^{N}={N-1 \over N} \sum _{m=1}^{N} I(A:X_m) - \sum _{m=1}^{N} I(M_m:X_m).$ $M_m$ indicates the different incompatible observables and $X_m$ stands for the memory particles for *m*-th measurement. In this uncertainty game, a multipartite quantum state $\rho _{AX_{1}...X_{N}}$ is shared by Alice and the others. Now, Alice measures one of the observables $M_m (m=1,2,...,N)$ on her quantum system. As Alice measures the observable $M_m$, the $X_m$’s task will be minimization his uncertainty about $M_{m}$.

The QMA-EUR has been realized to have potential applications in various quantum information processing tasks, such as quantum key distribution^5,43^, quantum metrology^44^, quantum cryptography^45,46^, quantum randomness^47,48^, entanglement witness^49,50^, EPR steering^51,52^, and so on.

Additionally, several authors have attempted to find relations between quantum correlations and EURs^53–76^. On the other hand, the monogamy of quantum correlation has broad application in quantum information^77–83^. In a recent study, Hu and Fan could obtain a new upper bound on quantum discord (QD) through bipartite QMA-EUR^53^. They also could extract an upper bound on shareability of QD.

In this paper, inspiring from^53^ and by using tripartite QMA-EUR, an upper bound on shareability of QD will be found. In the beginning, new relations for tripartite QMA-EUR are introduced. Then, it is shown that by using these relations, one could obtain a new upper bound for the shareability of QD. Also, it is shown that for specific states, the obtained relation could be considered as monogamy of QD. Finally, it is exhibited that the above procedure could be generalized to a multipartite system, in which an upper bound for the shareability of QD in a multipartite system is derived.

The paper has been organized as follows: In “Quantum discord”section, the QD will be defined as one of the measures of quantum correlation. In “Tripartite QMA-EUR and shareability of QD”section, the new relation for the tripartite QMA-EUR is expressed and also an upper bound for the shareability of QD is extracted. The results will be summarized in “Conclusion”section..

## Quantum discord

QD is another important concept within the field of quantum information. Considerable attention has been paid to QD due to its potential connection with other aspects of quantum information and beyond, including quantum communication, quantum computation, many-body physics, and open quantum dynamics (see^84^ for further details).

The concept of QD of a bipartite quantum system is defined in several ways which could be classified into two wide categories. One of these categories is based on measurement in any one of the subsystems, which will be used in our discussion.

QD is the difference between the total and the classical correlations^85,86^, namely,

11 $$\begin{aligned} D_A(\rho _{AB})=I(\rho _{AB})-J_A(\rho _{AB}), \end{aligned}$$

in which the subscript of $D_A(\rho _{AB})$ denotes that the measurement has been performed on the subsystem *A*. The total correlations in state $\rho _{AB}$ measured by the quantum mutual information (7) and the classical correlation $J_A(\rho _{AB})$, which is defined as

12 $$\begin{aligned} J_A(\rho _{AB}) = S(\rho _B)-min_{\Pi _i^A} S(\rho _{B|{\Pi _i^A}}), \end{aligned}$$

where $S(\rho _{B|{\Pi _i^A}})=\sum _i p_i S(\rho _{B|i})$ and the minimization is taken over all quantum measurements, ${\Pi _i^A }$, performed on the system *A*.

Recently, Hu and Fan have investigated a relation between QD and bipartite QMA-EUR^53^. Their consideration led to an improvement on the upper bounds for QD^53^. They also considered the effects of the bipartite QMA-EUR on the shearability of quantum correlation among different subsystems. With the use of the bipartite QMA-EUR, Hu and Fan found an upper bound on the shearability of QD among different parties of a composite system, which is given by^53^

13 $$\begin{aligned} D_A(\rho _{AB}) + D_A(\rho _{AC}) \le S(\rho _A) + \delta _T , \end{aligned}$$

in which $\delta _T = S(X|B) + S(Z|B) - q_{MU} - S(A|B)$. They showed that for any tripartite state $\rho _{ABC}$ with $S(\rho _A) = -S(A|BC)$, the above relation can be written as:

14 $$\begin{aligned} D_A(\rho _{AB}) + D_A(\rho _{AC}) \le D_A(\rho _{A:BC}) + \delta _T . \end{aligned}$$

This equation can be considered as the released version of the monogamy relation of QD. It applies to all tripartite pure states as well as to extended classes of mixed states^53^.

## Tripartite QMA-EUR and shareability of QD

In this section, inspired by Hu and Fan^53^, who obtained an upper bound on the shareability of QD among the constituent parties by using bipartite QMA-EUR, we are going to introduce a new upper bound on the shareability of QD by utilizing tripartite QMA-EUR.

### New lower bound for the tripartite QMA-EUR

Here, we introduce new tripartite QMA-EURs, which depend on the incompatibility of two quantum measurements, the strong subadditivity (SSA) inequality, the QD, and the classical correlations of a state shared between the observed system and quantum memories.

#### Theorem 1

For any tripartite state, the following equations hold

15 $$\begin{aligned} S(X|B) + S(Z|C)\ge & {} q_{MU} + {1 \over 2} \; \left[ S(A|B) + S(A|C) \right] + \mathrm{max} \{ O, \delta '^{3} \}, \end{aligned}$$

16 $$\begin{aligned} S(X|B) + S(Z|C)\ge & {} q_{MU} + {1 \over 2} \; \left[ S(A|B) + S(A|C) \right] + \mathrm{max} \{ O, \delta ''^{3} \}, \end{aligned}$$

where

17 $$\begin{aligned} \delta '^{3}= & {} {1 \over 2} \; \left\{ D_A(\rho _{AB}) + D_A(\rho _{AC}) - J_A(\rho _{AB}) - J_A(\rho _{AC}) \right\} , \end{aligned}$$

18 $$\begin{aligned} \delta ''^{3}= & {} \; \left\{ D_A(\rho _{AB}) + D_A(\rho _{AC}) - {1 \over 2} \left[ I(A:B) + I(A:C) \right] \right\} . \end{aligned}$$

#### Proof

The theorem is proved using the definition of classical correlation, QD, and tripartite QMA-EUR, Eq. (6). Regarding Eq. (6), one obtains

19 $$\begin{aligned} S(X|B) + S(Z|C)\ge & {} q_{MU} + {1 \over 2} \; \left[ S(A|B) + S(A|C) \right] + {1 \over 2} \left[ I(A:B) + I(A:C) \right] - I(X:B) - I(Z:C) \nonumber \\\ge & {} q_{MU} + {1 \over 2} \; \left[ S(A|B) + S(A|C) \right] + {1 \over 2} \left[ I(A:B) + I(A:C) - 2J_A(\rho _{AB}) - 2J_A(\rho _{AC}) \right] \nonumber \\= & {} q_{MU} + {1 \over 2} \; \left[ S(A|B) + S(A|C) \right] + {1 \over 2} \; \left\{ D_A(\rho _{AB}) + D_A(\rho _{AC}) - J_A(\rho _{AB}) - J_A(\rho _{AC}) \right\} . \end{aligned}$$

Note that in the second row of the above relation we have applied the definition of the classical correlation, $J_A(\rho _{AY}) = max_{\Pi ^A_i} I(P:Y)$, where $Y \in \{B,C\}$, and the fact that observables *X* and *Z* may not be necessarily the maximizing quantum measurements for $J_A(\rho _{AY})$, so that $J_A(\rho _{AB}) \ge I(X:B)$, and also $J_A(\rho _{AC}) \ge I(Z:C)$. In the last line of the above proof, the definition of QD has been used as well. The other equation of the theorem is proved by following the same procedure. $\square$

Also, one can utilize above procedure to obtain a relation for QMA-EUR for multipartite system.

#### Corollary 1.1

For any multipartite state, The equation below is hold

20 $$\begin{aligned} \sum _{i=1}^{N} S(M_i|X_i) \ge - \log _{2}(b) + {N-1 \over N} \sum _{i=1}^{N} S(A|X_i) + \mathrm{max}\{0,\delta '^{N} \}, \end{aligned}$$

where $$\delta '^{N}=\sum _{i=1}^{N} D_A(\rho _{AX_i})-{1 \over N} \sum _{i=1}^{N} I(A:X_i).$$

#### Proof

Regarding Eq. (9) and following the same method used in Theorem. 1, one can arrive at Eq.(20). $\square$

### Bounds on shareability of QD from the tripartite QMA-EUR

Applying the resulted obtained in the previous subsection and following the same approach presented in^53^, one can obtain an upper bound for the shareability of QD among different subsystems.

#### Theorem 2

For any tripartite state $\rho _{ABC}$, we have

21 $$\begin{aligned} \Delta _1 + \Delta _2 + S(\rho _A) \ge D_A(\rho _{AB}) + D_A(\rho _{AC}), \end{aligned}$$

where

$$\begin{aligned} \Delta _1 = S(X|B) + S(Z|C) - q_{MU} - {1 \over 2} \; \left[ S(A|B) + S(A|C) \right] , \end{aligned}$$

and

$$\begin{aligned} \Delta _2 = {-1 \over 2} \; \left[ S(A|B) + S(A|C) \right] . \end{aligned}$$

#### Proof

From Eq. (16), one arrives at

22 $$\begin{aligned} S(X|B) + S(Z|C) - q_{MU} - {1 \over 2} \; \left[ S(A|B) + S(A|C) \right] + {1 \over 2} \; \left[ I(A:B) + I(A:C) \right] \ge D_A(\rho _{AB}) + D_A(\rho _{AC}). \end{aligned}$$

Substituting the following relation

23 $$\begin{aligned} { 1 \over 2} \; \left[ I(A:B) + I(A:C) \right] = S(\rho _A) - {1 \over 2} \; \left[ S(A|B) + S(A|C) \right] , \end{aligned}$$

in Eq.(22), one comes to

24 $$\begin{aligned} S(X|B) + S(Z|C) - q_{MU} - {1 \over 2} \; \left[ S(A|B) + S(A|C) \right] + S(\rho _A) - {1 \over 2} \; \left[ S(A|B) + S(A|C) \right] \ge D_A(\rho _{AB}) + D_A(\rho _{AC}). \end{aligned}$$

Therefore, the theorem has been proved. $\square$

This is the main result of this work. As can be seen from Eq. (21), our relation contains three terms: $S(\rho _A)$ which implies the entropy of the subsystem *A*, $\Delta _1$ that depends on the tripartite QMA-EUR, and $\Delta _2$ that is related to the SSA inequality. From the tripartite EUR, we can find $\Delta _1 \ge 0$, and from the SSA inequality, it is realized that $\Delta _2 \le 0$.

Let us now compare our upper bound (21) with Hu and Fan upper bound (13). Due to the fact that the following two relations

$$\begin{aligned} S(X|B) + S(Z|B) \ge q_{MU} + S(A|B) , \end{aligned}$$

and

$$\begin{aligned} S(X|B) + S(Z|C) \ge q_{MU} , \end{aligned}$$

are equivalent^5^, it is realized that our bound has one extra term as $-\left( S(A|B)+S(A|C) \right)$. From SSA inequality, one finds that the term is negative, i.e. $- \left( S(A|B)+S(A|C) \right) \le 0$. Therefore, our bound is tighter than that of Hu and Fang. However, for states that SSA inequality is saturated, the upper bound of Eq. (21) is equivalent to the bound of Eq. (13) obtained by Hu and Fan^53^.

It is interesting to note that for all tripartite pure and some special mixed tripartite states, our upper bound is a released version of the monogamy relation of QD^87–90^.

#### Corollary 2.1

For any tripartite state $\rho _{ABC}$ with $S(A) = - S(A|BC)$, one has

25 $$\begin{aligned} D_A(\rho _{AB}) + D_A(\rho _{AC}) \le D_A(\rho _{A:BC}) + \Delta _1 + \Delta _2. \end{aligned}$$

#### Proof

The outline of the proof is similar to what we have in^53^. Due to the fact that $D_A(\rho _{A:BC}) = S(\rho _A)$ and $S(\rho _A) = -S(A|BC)$, it is realized that Eq. (25) is valid for all tripartite pure states. As stated in^91^, under a specific condition, the relation $S(\rho _A) = -S(A|BC)$ is reliable even for a mixed state $\rho _{ABC}$. The relation is true for a mixed state if and only if for the Hilbert space ${\mathscr {H}}_{BC}$ we have a factorization ${\mathscr {H}}_{BC} = {\mathscr {H}}_{(BC)^L} \otimes {\mathscr {H}}_{(BC)^R}$ in which $\rho _{ABC} = |\psi \rangle _{A(BC)^L} \langle \psi | \otimes \rho _{(BC)^R}$. For this case, it is obtained that $D_A(\rho _{A:BC}) = D_A(|\psi \rangle _{A(BC)^L}) = S(\rho _A)$. $\square$

Our results should have several useful applications in the areas of quantum information theory. One of the consequences of our inequality (25) is that, if for tripartite pure state $| \psi \rangle _{ABC}$ one finds two observables *X* and *Z* that saturate $S(X|B) + S(Z|C) \ge q_{MU}$, then it could be stated that we have the sufficient condition for the monogamy QD. The generalized Greenberger-Horne-Zeilinger (GHZ) state could be implied as one of the examples of the situation.

### Examples

To clarify the above-mentioned results, four examples are considered. For the first two examples, we consider pure states, however for the next two examples, mixed states are investigated. In these examples, the observables that are measured on the part *A* of quantum states are assumed to be the Pauli matrices $X = \sigma _{1}$ and $Z = \sigma _{3}$.

#### Generalized GHZ state

**Figure 1:**
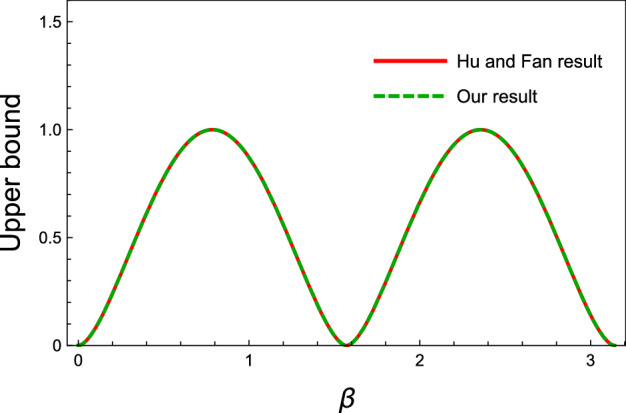
Different upper bounds on the shareability of QD for the state in Eq. (26), versus the state’s parameter $\beta$. The figure vividly shows that our upper bound and that of Hu and Fan will be the same. It is due to this point that for any pure state, as Eq.(26), the difference between our upper bound and Hu and Fan upper bound will be eliminated.

First, let us consider the generalized GHZ states which have the form

26 $$\begin{aligned} \vert gGHZ\rangle = \cos \beta \vert 000\rangle + \sin \beta \vert 111\rangle , \end{aligned}$$

where $\beta \in \left[ 0,2\pi \right)$. In Fig. 1, different upper bounds of the shareability of QD for these states are plotted versus the parameter $\beta$. As it was expected, the obtained upper bound (21) coincides with Hu and Fan upper bound (13).

**Figure 2:**
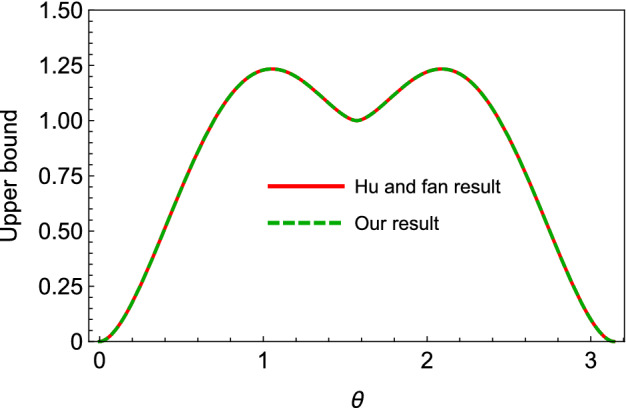
Different upper bounds on the shareability of QD for the state in Eq. (27), versus the state’s parameter $\theta$, where $\phi =\pi /4$. Since the state is pure, our upper bound will be the same as the upper bound of Hu and Fan, which is plotted in the figure.

#### Generalized W state

As the second example, consider the following generalized *W* state:

27 $$\begin{aligned} \vert gW \rangle = \sin \theta \cos \phi \vert 100\rangle + \sin \theta \; \sin \phi \vert 010\rangle + \cos \theta \vert 001\rangle , \end{aligned}$$

where $\theta \in \left[ 0,\pi \right]$ and $\phi \in \left[ 0,2\pi \right)$. Same as the previous case, it is realized that for this state, the obtained upper bound (21) is exactly the same as that of Hu and Fan (13); shown in Fig. 2.

**Figure 3:**
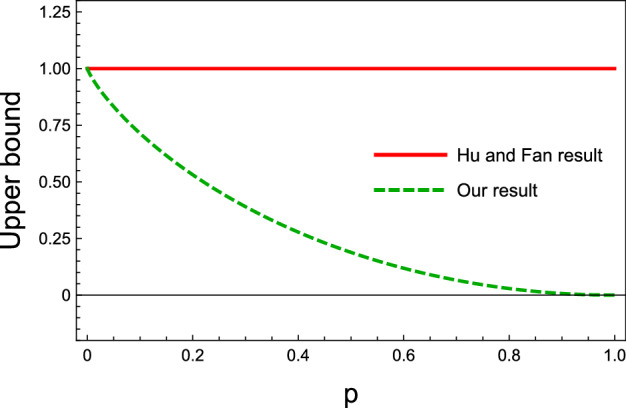
Different upper bounds on the shareability of QD for the state in Eq. (28), versus the state’s parameter *p*, where $0 \le p \le 1$. The solid-red curve stands for Hu and Fan upper bound and the dashed-green curve indicates our upper bound. At $p=0$, the state is pure, and our bound coincides with Hu and Fan upper bound; as it was expected for a pure state. It is realized that for $0<p< 1$, our upper bound is tighter than that of Hu and Fan, and for $p=1$ it reaches zero which shows that at this point there is no quantum correlation.

**Figure 4:**
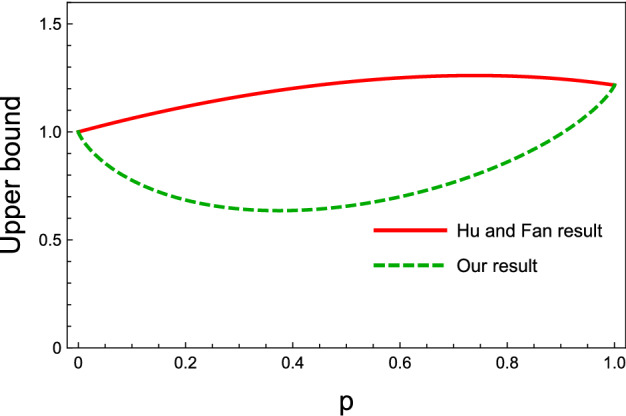
Different upper bounds on the shareability of QD for the state in Eq. (29), versus the state’s parameter *p*, where $0 \le p \le 1$. The solid-red curve stands for Hu and Fan upper bound and the dashed-green curve indicates our upper bound. Since for $p=0$ and $p=1$, there is a pure state, our bound completely coincides with that of Hu and Fan. However, the curves illustrate that for $0<p<1$, our obtained upper bound is tighter than that of Hu and Fan.

#### Werner-GHZ state

As an another example, we consider Werner-GHZ state, defined as

28 $$\begin{aligned} \rho _{w}=(1-p) \vert GHZ \rangle \langle GHZ \vert + \frac{p}{8}{\mathbf {I}}_{ABC}, \end{aligned}$$

where $\vert GHZ \rangle = 1/\sqrt{2}(\vert 000 \rangle + \vert 111 \rangle )$ is the GHZ state, and $0 \le p \le 1$. In Fig. 3, the upper bounds of the shareability of QD for this state are plotted versus the parameter *p*. As can be seen, Hu and Fan upper bound (13) is constant as a function of the parameter *p*, whereas our upper bound (21) is tighter and also it decreases by enhancement of *p*, and reaches zero at $p=1$. From physical point of view, this is an acceptable result because at $p=1$ we have the maximally mixed state and there is no quantum correlation. This physical feature is illustrated properly in our upper bound, however, the Hu and Fan bound does not exhibit such a feature.

#### A mixed three-qubit state

As the last example, let us consider a state of the following form

29 $$\begin{aligned} \rho =(1-p)\vert GHZ \rangle \langle GHZ \vert + p\vert W \rangle \langle W \vert , \end{aligned}$$

where $0 \le p \le 1$ is a real number and the usual $\vert W \rangle$ state is defined as

$$\begin{aligned} \vert W \rangle= & {} \frac{1}{\sqrt{3}}(\vert 001 \rangle + \vert 010 \rangle + \vert 100 \rangle ). \end{aligned}$$

In Fig. 4, the upper bounds of the shareability of QD for the state in Eq. (29) are plotted versus the parameter *p*. According to the figure, it is realized that at $p=0$ and $p=1$, our upper bound coincides with the upper bound of Hu and Fan because there are pure states. However, for $0<p<1$ where the states are mixed, our upper bound is tighter than that of Hu and Fan.

### Generalization

An implication of the presented method and results is that they could be generalized to obtain a constraint on the shareability of the QD among different parties of a $(N+1)$-partite states. By utilizing the multipartite uncertainty relation with quantum memory, it is possible to find an upper bound for the shareability of multipartite QD. This will be presented in the following theorem.

#### Theorem 3

For any $N+1$-partite state, we have

30 $$\begin{aligned} \Delta _1^N + \Delta _2^N + S(\rho _A) \ge \sum _{i=1}^{N} D_A(\rho _{AX_i}), \end{aligned}$$

in which

31 $$\begin{aligned} \Delta _1^N= & {} \sum _{i=1}^{N} S(M_i|X_i) + \log _2(b) - {N-1 \over N} \sum _{i=1}^{N} S(A|X_i), \end{aligned}$$

32 $$\begin{aligned} \Delta _2^N= & {} {-1 \over N} \sum _{i=1}^{N} S(A|X_i). \end{aligned}$$

#### Proof

Regarding the Eq. (20), one has

33 $$\begin{aligned} \sum _{i=1}^{N} S(M_i|X_i) + \log _{2}(b) - {N-1 \over N} \sum _{i=1}^{N} S(A|X_i) + {1 \over N} \sum _{i=1}^{N} I(A:X_i) \ge \sum _{i=1}^{N} D_A(\rho _{AX_i}) , \end{aligned}$$

Applying the relation below

34 $$\begin{aligned} S(\rho _A) = {1 \over N} \; \left[ \sum _{i=1}^{N} S(A|X_i) + \sum _{i=1}^{N} I(A:X_i) \right] , \end{aligned}$$

one comes to

35 $$\begin{aligned} \sum _{i=1}^{N} S(M_i|X_i) + \log _2(b) - {N-1 \over N} \sum _{i=1}^{N} S(A|X_i) + S(\rho _A) - {1 \over N} \sum _{i=1}^{N} S(A|X_i) \ge \sum _{i=1}^{N} D_A(\rho _{AX_i}) . \end{aligned}$$

The above equation could be rewritten as

36 $$\begin{aligned} \Delta _1^N + \Delta _2^N + S(\rho _A) \ge \sum _{i=1}^{N} D_A(\rho _{AX_i}). \end{aligned}$$

$$\square $$

Now, let us consider the above result for a four-partite state, i.e. $N=3$. For this case, Eq. (30) is rewritten as

37 $$\begin{aligned} \Delta _1^3 + \Delta _2^3 + S(\rho _A) \ge D_A(\rho _{AB}) + D_A(\rho _{AC}) + D_A(\rho _{AD}), \end{aligned}$$

where the quantity $\Delta _1^3$ is given by

38 $$\begin{aligned} \Delta _1^3 = S(M_1|B) + S(M_2|C) + S(M_3|D) + \log _2(b') - {2 \over 3} \; \left[ S(A|B) + S(A|C) + S(A|D) \right] , \end{aligned}$$

and

39 $$\begin{aligned} b' = max_k \left\{ \sum _j max_i [|\langle u_i^1 | u_j^2 \rangle |^2] \; |\langle u_j^3 | u_k^3 \rangle |^2 \right\} , \end{aligned}$$

in which $|u_i^1 \rangle$, $| u_j^2 \rangle$, and $| u_k^3 \rangle$ are the eigenstates of the three observables $M_1$, $M_2$, and $M_3$, respectively. The other quantity $\Delta _2^3$ is read as

40 $$\begin{aligned} \Delta _2^3 = {-1 \over 3} \; \left[ S(A|B) + S(A|C) + S(A|D) \right] . \end{aligned}$$

Assume there is a four-partite state $\rho _{ABCD}$, where the particles A, B, C, and D are respectively sent to Alice, Bob, Charlie, and David. Then, Alice does a measurement on her quantum system with one of the three observables $M_m$ (where $m=1,2,3$) and informs the other about her choice of measurement. If Alice measures $M_1$, it is Bob’s duty to minimize his uncertainty about $M_1$. If $M_2$ is measured by Alice, it is Charlie’s task to minimize his uncertainty about $M_2$. And for the last case, if $M_3$ is measured by Alice, it is David’s task to minimize his uncertainty about $M_3$.

## Conclusion

There are many applications for the tripartite QMA-EUR in quantum information theory; quantum key distribution could be addressed as one of these applications. In the presented work, we introduced another application of tripartite QMA-EUR. It was discussed that using tripartite QMA-EUR, one could obtain an upper bound for the shareability of QD. Our bound includes three terms in which one is related to the entropy of the subsystem that is being measured. The second term is related to the tripartite QMA-EUR, and the third term implies the SSA inequality. In another word, our bound relates tripartite QMA-EUR, SSA inequality, and QD which are known as three important features of quantum information. A comparison was made between the obtained upper bound and Hu and Fan upper bound for some states such as the Werner-GHZ state and a mixed three-qubit state. Results show that our upper bound is tighter than that obtained by Hu and Fan^53^.

The obtained bound could be applicable in the field of quantum information. This relation can be converted into the monogamy of QD for certain tripartite quantum states. The result indicates that for a tripartite pure state if one can find two observables *X* and *Z* that saturate $S(X|B) + S(Z|C) \ge q_{MU}$, then, a sufficient condition for the monogamy of QD is provided.

Furthermore, the work can be generalized to obtain an upper bound on the shareability of QD for multipartite states, indicating that quantum correlations cannot be freely shared.
